# Evolutionary Analyses of Entire Genomes Do Not Support the Association of mtDNA Mutations with Ras/MAPK Pathway Syndromes

**DOI:** 10.1371/journal.pone.0018348

**Published:** 2011-04-19

**Authors:** Alberto Gómez-Carballa, María Cerezo, Emilia Balboa, Claudia Heredia, Lidia Castro-Feijóo, Itxaso Rica, Jesús Barreiro, Jesús Eirís, Paloma Cabanas, Isabel Martínez-Soto, Joaquín Fernández-Toral, Manuel Castro-Gago, Manuel Pombo, Ángel Carracedo, Francisco Barros, Antonio Salas

**Affiliations:** 1 Unidade de Xenética, Departamento de Anatomía Patolóxica e Ciencias Forenses, and Instituto de Medicina Legal, Facultade de Medicina, Universidad de Santiago de Compostela, Santiago de Compostela, Galicia, Spain; 2 Unidad de Medicina Molecular, Fundación Pública Galega de Medicina Xenómica, CIBERER, Santiago de Compostela, Galicia, Spain; 3 Unidad de Endocrinología Pediátrica, Crecimiento y Adolescencia, Departamento de Pediatría, Hospital Clínico Universitario y Universidad de Santiago de Compostela, Santiago de Compostela, Galicia, Spain; 4 Servicio de Endocrinología Infantil, Hospital de Cruces, Barakaldo, Basque Country, Spain; 5 Unidad de Neurología Pediátrica, Departamento de Pediatría, Hospital Clínico Universitario y Universidad de Santiago de Compostela, Santiago de Compostela, Galicia, Spain; 6 Unidad de Cardiología Infantil, Departamento de Pediatría, Hospital Clínico Universitario de Santiago de Compostela, Santiago de Compostela, Galicia, Spain; 7 Genética, Hospital Central de Asturias, Oviedo, Asturias, Spain; University of Glasgow, United Kingdom

## Abstract

**Background:**

There are several known autosomal genes responsible for *Ras*/*MAPK* pathway syndromes, including Noonan syndrome (NS) and related disorders (such as LEOPARD, neurofibromatosis type 1), although mutations of these genes do not explain all cases. Due to the important role played by the mitochondrion in the energetic metabolism of cardiac muscle, it was recently proposed that variation in the mitochondrial DNA (mtDNA) genome could be a risk factor in the Noonan phenotype and in hypertrophic cardiomyopathy (HCM), which is a common clinical feature in Ras/MAPK pathway syndromes. In order to test these hypotheses, we sequenced entire mtDNA genomes in the largest series of patients suffering from *Ras*/*MAPK* pathway syndromes analyzed to date (*n* = 45), most of them classified as NS patients (*n* = 42).

**Methods/Principal Findings:**

The results indicate that the observed mtDNA lineages were mostly of European ancestry, reproducing in a nutshell the expected haplogroup (hg) patterns of a typical Iberian dataset (including hgs H, T, J, and U). Three new branches of the mtDNA phylogeny (H1j1, U5b1e, and L2a5) are described for the first time, but none of these are likely to be related to NS or *Ras*/*MAPK* pathway syndromes when observed under an evolutionary perspective. Patterns of variation in tRNA and protein genes, as well as redundant, private and heteroplasmic variants, in the mtDNA genomes of patients were as expected when compared with the patterns inferred from a worldwide mtDNA phylogeny based on more than 8700 entire genomes. Moreover, most of the mtDNA variants found in patients had already been reported in healthy individuals and constitute common polymorphisms in human population groups.

**Conclusions/Significance:**

As a whole, the observed mtDNA genome variation in the NS patients was difficult to reconcile with previous findings that indicated a pathogenic role of mtDNA variants in NS.

## Introduction

Noonan syndrome (NS) was first described by Noonan and Ehmke [1]. It refers to a pleiomorphic autosomal dominant disorder with short stature, facial dysmorphia, a webbed neck and heart defects, and its prevalence is about one in 1000–2500 live births [2]. Cardiovascular diseases including valvular pulmonary stenosis, atrial septal defect and hypertrophic cardiomyopathy (HCM) are generally observed in 50–80% of the patients, with HCM being one of the most common cardiac abnormalities in these patients [3], [4], [5], [6].

Several germ line gain-of-function mutations in several RAS pathway members, including *PTPN11* (which encode tyrosine phosphatase SHP-2), *KRAS*, *SOS1*, *BRAF*, and *RAF1*, *SHOC2*, *MEK1* (alias *MAPP2K1*) have been identified as being responsible for NS [7], [8], [9], [10], [11], [12]. It has been suggested that nuclear DNA (nDNA) mutations in *PTPN11* account for about ∼50% of cases [13]. Mutations in *KRAS*, *SOS1*, and *RAF1* make up ∼1–2%, ∼20%, and 3–5% of NS cases without *PTPN11* mutations, respectively [14]. When combined, all the above mentioned nuclear genes would account for 70–85% of NS cases [15]. Thus far, seven genes have been causally related to NS but also to other closely related conditions, including LEOPARD syndrome and Noonan-like syndrome. Germline mutations in a subgroup of those genes and other genes encoding signal transducers participating in the same pathway (*HRAS*, *KRAS*, *NF1*, *SPRED1*, *BRAF*, *MEK1* and *MEK2*, alias *MAP2K2*) have been identified to be implicated in other clinically related disorders such as Costello syndrome ore neurofibromatosis type 1 [16]. Some authors proposed to group these developmental diseases in a single family of disorders, which has been termed the neurocardiofacialcutaneous syndrome (NCFCS) family [16], the *Ras*/*MAPK* pathway syndromes or RASopathies [17].

The search for new causal genes responsible for *Ras*/*MAPK* pathway syndromes has motivated many authors to explore the potential role of mtDNA mutations in NS and HCM based on the assumption that the mitochondrion plays an essential role in the energetic metabolism of cardiac muscle. Thus, recently, Dhandapany et al. [18] reported nine mtDNA mutations in a Noonan Indian patient suffering hypertrophic obstructive cardiomyopathy. According to the authors, “*Our case forms the first report, which emphasizes the importance of mtDNA mutations in Noonan syndrome and extends the scope for mitochondrial related syndromes*” (p. 287) [18]. The study of Dhandapany et al. was based on the analysis of only one patient's complete mtDNA genome, and the full set of results was not reported by the authors: only a list of nine mutations observed in the patient's mtDNA genome was reported. Eight of these mutations were reported as novel, a finding that was interpreted by the authors as follows: “*the identification of these mutations indicates that mutations in mtDNA may account for a significant portion of genetic etiology in Noonan syndrome*” (p. 287) [18]. A year before the appearance of the study by Dhandapany's et al. [18], Prasad et al. [19] claimed that they had observed six novel mutations in an Indian HCM patient. Both studies attributed the pathogenic condition to their presumable “novel” variants without any further scientific support. The misconception of “novelty” being synonymous with “causality” for mtDNA variants is unfortunately all too common in medical literature. And this is particularly problematic in mtDNA studies due to the fact that the mtDNA molecule is highly variable in human populations; in fact, a large proportions of both rare and common variations in populations still remain to be discovered and are consequently unrecorded in databases. As discussed in Bandelt et al. [20], “…*An observed mtDNA mutation or polymorphism is novel if it has not been observed before; that is, it has not been reported in the literature before or cannot be found in other publicly available source. This, however, is not the manner in which the novelty of mtDNA mutations is perceived and treated in practice by the working human geneticist*.” (p. 1073) [20]. Novelty is almost always operationally defined by searching for mtDNA in the main reference mtDNA database in the field, namely, MITOMAP (http://www.mitomap.org/cgi-bin/mitomap/search.pl) [20]. However, MITOMAP, although useful for many medical applications, is deficient in few aspects [20], and has therefore been interpreted as a risk factor in medical studies [21], [22].

Other recent articles have contributed to the debate on the presumable association of mtDNA variants or haplogroups (hgs) with NS or HCM. For example, Castro et al. [23] claimed to have found an association between hg T and NS patients of European ancestry, while Rani et al. [24] reported a presumable association between hg R and NS cases in a very small cohort of seven Indian patients.

The present study was motivated by this controversy. We conducted a sequencing study of the whole mtDNA genome in a total of 45 patients suffering *Ras*/*MAPK* pathway syndromes; most of them were NS patients (93%). About 11% of the patients were also affected by HCM. This is the largest cohort of *Ras*/*MAPK* pathway syndromes and Noonan patients who were analyzed for mtDNA variations by far. An evolutionary approach was carried out in order to assist the interpretation of variations found in NS patients. This approach was shown to be very useful in a previous study dealing with the analysis of mtDNA variation in asthenozoospermic males [25]. The alternative method of using a mtDNA case-control population study would require a much larger sample size, which is unfeasible for rare traits such as NS [26], [27]. We aimed to address several issues in the present study: (i) to evaluate whether *Ras*/*MAPK* pathway syndromes (with especial focus in NS patients) cluster in particular hgs by comparing the data with data available from human populations of the same ancestry, (ii) to identify mutations that could explain the clinical phenotypes of our patients, (iii) to evaluate whether replacement substitutions accumulate to a greater extent in patients with respect to expectations derived from control individuals (represented by the worldwide phylogeny based on more than 7800 entire genomes); (iv) to see whether tRNA mutations are more prevalent in patients than in control individuals, and (v) to examine recurrent, private and heteroplasmic mutations for patterns that could explain the clinical phenotypes. Moreover, previous findings claiming an association between mtDNA mutations or hgs and NS are discussed here for the first time in view of the present evolutionary evidence.

## Methods

### Ethics statement

Written informed consent was required from all patients. Analysis of entire mtDNA genomes in patient samples was approved by the Ethical committee of the University of Santiago de Compostela. The study conforms to the Spanish Law for Biomedical Research (Law 14/2007- 3 of July).

### Sample collection and DNA extraction

**Figure 1:**
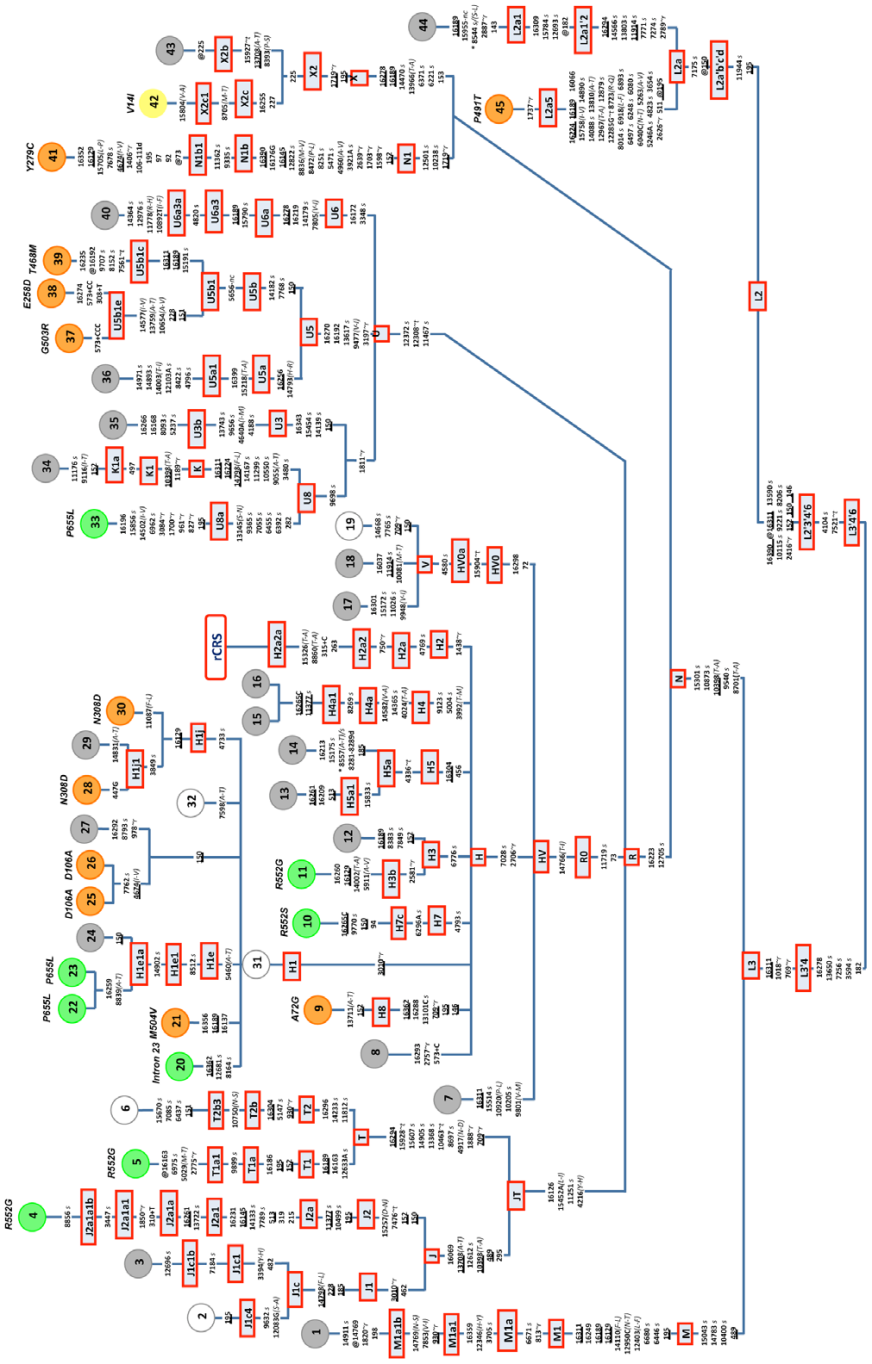
Maximum parsimony tree of 45 entire mtDNA genomes of patients suffering Ras/MAPK pathway syndromes. The mutations are displayed along branches; the variant nomenclature is refered to was taken from the rCRS [30]. All mutations are transitions unless a suffix specifies a transversion (A, C, G, T), a deletion (d), an insertion (+), a synonymous substitution (*s*), a mutational change in tRNA (*-t*), a mutational change in rRNA (*-r*), stop codon (*-stp*), non-coding variant located in the mtDNA coding region (*-nc*) or amino acid replacement (indicated in round brackets). Recurrent mutational events are underlined. A prefix indicates a back mutation (@) or a position that is located in an overlapping region shared by two genes (*). Several mutational hotspot variants were not considered for phylogenetic reconstruction and therefore were eliminated from the tree; these included variants at the homopolymeric tracks around position 310, the microsatellite at m.523–524 d (aka m.522–523 d), the transversion m.16182A>C, m.16183A>C, m.16193+1C(C), m.16519T>C, and length or point heteroplasmies. Codes of the samples are indicated in colored circles at the terminal branches of the phylogeny: green indicates a mutation on gene *SOS1*, orange indicates a mutation on *PTPN11*, yellow indicates a mutation on *KRAS*, grey indicates lack of mutations on genes *SOS1*, *PTPN11*, *KRAS*, and *RAF*, and white indicates that data is not available for that sample.

Blood samples were collected from all patients anonymously. A total of 45 samples were recruited for the present study. Our samples include 42 NS cases, two LEOPARD syndrome patients, and one neurofibromatosis type 1 patient. Note however, that the NS is clinically variable and a genetically heterogeneous developmental disorder; therefore our collection of patients was grouped more generally as patients suffering *Ras*/*MAPK* pathway syndromes. Among the 45 patients recruited, we included three pairs of brothers (namely, patients #15 and #16, #22 and #23, #25 and #26; see Figure 1). The DNA was extracted following standard phenol-chloroform protocols. Table 1 summarizes the clinical-pathological characteristics of our patients.

**Table 1:** Clinico-pathological characteristics of the patients; numbers indicate percentages of the total sample.

Phenotype	Sub-phenotype/Sub-classification	%
**Facies**		
	typical	40.0
	suggestive	28.9
**Cardiac features**		
	typical ECG	15.6
	hypertrophic cardiomyopathy	11.1
	pulmonary valvular stenosis	26.7
	septal isolated defects	2.2
	bivalve aorta	2.2
	pulmonary artery dysplasia idiopathic dilatation	4.4
	septal atrial defects	2.2
**Height**		
	percentile<3	35.6
	percentile<10	17.8
**Thoracic abnormalities**		
	pectus escavatum/carinatum	26.7
	broad thorax	33.3
**Family history**		
	first degree suggestive	8.9
	first degree definitive	13.3
**Others**		
	mental retardation	8.9
	cryptorchidism	20.0
	lymphatic dysplasia	6.7
**Mutation in nuclear genes**		
	*PTPN11*	24.4
	*KRAS*	2.2
	*SOS1*	17.8
	*RAF*	0.0
	Total	44.4

### Complete genome sequencing

The DNA from all patients was sequenced for the entire mtDNA molecule. We followed the sequencing protocols used by Álvarez-Iglesias et al. [28], which are briefly described here. The primers used for Polymerase Chain Reaction (PCR) amplification and sequencing were reported previously [29]. Polymerase Chain Reaction was performed in 10 µL of the reaction mixture, containing 4 µL of PCR Master Mix (Qiagen; Hilden, Germany), 0.5 µL 1 µM of each primer, 1 µL sample template and 4 µL of water. This PCR was carried out in a 9700 Thermocycler (AB) with one cycle of 95°C for 15 min and then 35 cycles of 94°C for 30 s, 58°C for 90 s and 72°C for 90 s with a full extension cycle of 72°C for 10 min. The sequencing reaction was performed in 11.5 µL of the reaction mixture, containing 2.5 µL of sequencing buffer (5X), 0.5 µL of BigDye Terminator v3.1 Cycle Sequencing Kit (Applied Biosystems), 1 µL of the corresponding primer (final concentration was 1 µM), 3 µL of the purified PCR product and water up to 11.5 µL. The automatic mtDNA sequencing was carried using capillary electrophoresis ABI3730 (Applied Biosystems).

### Nomenclature and quality control

The revised Cambridge Reference Sequence or rCRS [30] was referred to for mtDNA variations. Haplogroup nomenclature was based on previous studies [28], [31], [32], [33], [34], [35]; the reference phylogeny is being updated by the project Phylotree [36]; see mtDNA tree Build 11 (7 Feb 2011) (http://www.phylotree.org/). In order to reduce the impact of sequencing artefacts [37], [38], [39], [40] we followed the phylogenetic procedures described in [40], [41], [42] which basically consisted of using the mtDNA worldwide tree as a reference to avoid artefactual profiles and documentation errors in mtDNA sequences and SNP genotypes as much as possible. This approach aimed to detect artificial patterns of mtDNA variations that significantly differed from the expected natural ones.

### Statistical analysis

Counts of different types of mutational changes were carried out as in Elson et al. [43]. Pearson's chi-square test was applied to 2×2 contingency tables. A maximum parsimony tree was built using the genetic information from the entire mtDNA molecule (excluding the fastest mutational variants). Relative positional mutation rates were taken from Soares et al. [44].

All of the statistical analyses were carried out individually for carriers and non-carriers of nDNA mutations. However, the amount of mutations accumulated in both groups for the different mutational categories (nonsynonymous, synonymous, tRNA, and recurrent, among others) was statistically non-significant in all cases (Pearson's chi-square test, *p*-value>0.05). Therefore, given that mutational patterns were almost similar for carriers and non-carriers of nDNA mutations, the figures and tables presented in the main text refer to the total sample size of patients. However, for the sake of clarity, the analyses carried out separately for carriers and non-carriers are presented in Supplementary Figures S1, S2, S4, and S5. Analyses were also carried out for NS patients alone, and as expected, the results were virtually the same than those obtained for the whole sample (data not shown) given that NS cases represented 93% of the sample.

The dataset of mtDNA profiles reported by Álvarez-Iglesias et al. [28] representing a typical northern Iberian population was used as a control group for haplogroup frequency comparisons with patients.

## Results

### Nuclear mutations and clinical features

Seven genes (*PTPN11*, *SOS1*, *KRAS*, *RAF1*, *BRAF*, *SHOC2* and *MEK1*, alias MAP2K1) have been causally related to NS and closely related conditions (including LEOPARD syndrome) and clinically related disorders (e.g. neurofibromatosis type 1) [2]. All of our patients were screened for mutations in nuclear DNA [45; and author's unpublished data]. About 44% of them carried nDNA mutations; in particular, most of them (∼24%) harboured mutations on the *PTPN11* gene, and some of them on *SOS1* (∼18%), and *KRAS* (∼2%) (Figure 1 and Table 1). No mutations on the *RAF1* gene were identified in negative cases of *PTPN11*, *SOS1*, and *KRAS*. Patients #39 and #41 were posteriorly diagnosed with suffering from LEOPARD syndrome, and in fact, they carried the characteristic mutations on genes *PTPN11* (Figure 1, Supplementary Table S1). Patient #35 suffered from neurofibromatosis type 1 syndrome (NF1) and also carried a 6 Mb deletion at 17q11-12. Nearly half of the patients suffered from cardiopathies, especially HCM (∼11%) and pulmonary valvular stenosis (∼27%). The other clinico-pathological characteristics of the patients are summarized in Table 1.

### Phylogeography and phylogeny of patient mtDNA genomes

Entire, complete genomes were obtained for our cohort of 45 patients (Supplementary Figure S1 and Table S1). Patient mtDNA lineages were allocated to their corresponding hgs: most of them were of European ancestry, and therefore included representatives of the main clades, H, V, U, K, T, J, X and N1b (Figure 1). Two patients (patients #37 and #38; Figure 1) belonged to a still unknown branch of haplogroup U5, here referred to as U5b1e, whereas two other patients (patients #28 and #29; Figure 1) belonged to a new branch within H1j, here referred to as H1j1. Two additional profiles belonged to the typical sub-Saharan hg L2 [46], [47], [48]. One of them fell within the sub-branch L2a1. The other one (patient #45) described a novel branch of the L2 phylogeny referred to here as L2a5; it shared a transition at position 7175 and a reversion at site 150 with hg L2a (Figure 1), and most of the variants were also shared with another entire genome uploaded in GenBank under accession number HM596745. Another mtDNA belonged to the predominantly North African clade M1, in particular to the branch M1a1b, a lineage very closely related to the Sardinian-specific M1a1b1 sub-clade [49]. The proportion of non-European lineages in our patients mirrored the proportion expected in a typical sample of healthy individuals from northern Iberia [28], [50], [51] (see Supplementary Figure S1). Furthermore, the distribution of hgs was almost identical in carriers and non-carriers of nDNA mutations (Supplementary Figure S1). The newly discovered branches of the mtDNA phylogeny (H1j1, U5b1e, L2a5) did not carry features indicating an association with the NS phenotype or more generally, with Ras/MAPK pathway syndromes (see more analyses below).

We did not observe a correlation between the mtDNA hg lineages of patients and whether they were positive or negative for nuclear gene mutations (Figure 1); in other words, mutations in nuclear genes do not seem to be correlated with the mtDNA background of an individual. For instance, within hg H, only half of the patients carried mutations on nuclear genes and almost all of the carriers of nuclear mutations belonged to different sub-branches of hg H.

In addition, patients negative for nDNA mutations showed different mtDNA backgrounds. Therefore, there is no evidence to indicate that basal mutations from the mtDNA tree are involved in Ras/MAPK pathway syndromes.

**Figure 2:**
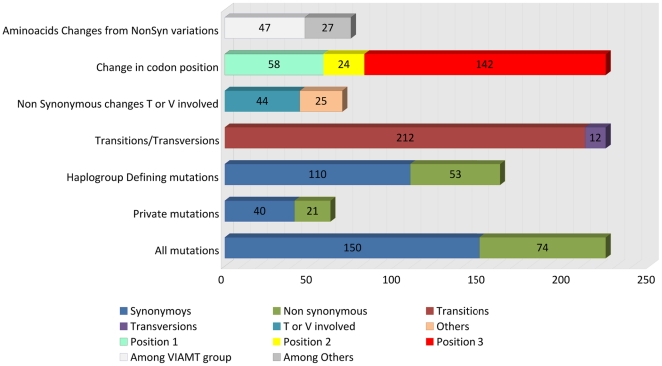
Summary of the main features regarding different types of mtDNA changes in patients.

The phylogenetic tree in Figure 1 shows a total of 224 substitution events occurring at the coding region (sequence range 577–16023) of the mtDNA genomes of the patients analyzed. Ten of these were recurrent mutations (Table 2); of these, four were nonsynonymous changes, and two of them involved the threonine codon. The latter fits well with the estimation of Kivisild et al. [52], indicating that most of the nonsynonymous substitutions involved this codon. Nonsynonymous changes are more common in the amino acid groups V, I, A, M, and T (VIAMT group; see Figure 2), and most of the changes were between neutral apolar amino acids (Supplementary Figure S2), as previously noted by Pereira et al. [53] in natural populations, suggesting that these changes in the VIAMT group are more easily tolerated than other amino acid changes.

**Table 2:** Homoplasmic position in the coding region mtDNAs of Ras/MAPK pathway syndromes patients.

Recurrent position	Sample ID^1^	Nucleotide change	Gene Location	Syn/Nonsyn (aa substitution)	Hg	non-hg	Soares et al. 2
709	#5*, #6, #9*, #19	G-A	12S rRNA	*	3 (T, H8)	1	59
930	#1, #6	G-A	12S rRNA	*	2 (M1a1b, T2b)	0	5
1719	#41*_,_#42*, #43	G-A	16S rRNA	*	3 (N1, X2)	0	31
3010	#2, #3, #20*, #21*, #22*, #23*, #24; #25*,#26*, #27*, #28*, #29; #30*, #31, #32	G-A	16S rRNA	*	13 (H1, J1)	0	19
4674	#41*; #25*, #26*	A-G	ND2	Nonsyn (I-V)	0	2	2
10398	#1, #2, #3, #4*, #34, #44, #45*	A-G	ND3	Nonsyn (T-A)	7 (J, K1, N)	0	18
11377	#4*, #15, #16	G-A	ND4	Syn	1 (J2a)	1	9
11914	#18, #44	G-A	ND4	Syn	1 (L2a1'2)	1	37
13708	#2, #3, #4*, #43,	G-A	ND5	Nonsyn(A-T)	4 (X2b, J)	0	24
14798	#2, #3, #34	T-C	CYT B	Nonsyn(F-L)	3 (J1c, K)	0	7

NOTE.

1 Starst identified samples carrying nDNA mutations;

2 Number of mutation hits in a worldwide phylogeny as recorded in Soares et al. [44].

The percentages of changes at the first, second and third positions of the codons were ∼26%, ∼11%, and ∼63%, respectively (Figure 2). This pattern resembles the one obtained in the set of complete genomes analyzed by Pereira et al. [53], namely: 24%, 13%, and 63%. This finding confirmed that while the third position is under weaker evolutionary pressure than the first and the second positions, there is a significant bias against mutations at the second codon position.

The amino acid T and V codons were more frequently hit by nonsynonymous changes than other amino acid codons (Figure 2), in a proportion 1.76∶1; a similar figure to the one obtained by Kivisild et al. [52], namely: 1.7∶1.

The ratio of transitions:transversion was 17.6∶1 (Figure 2); this ratio also fits well with the one obtained by Pereira et al. [53], which was 22.2∶1, when considering polymorphism over 0.1%, which suggests the action of negative selection against transversion. The spectrum of transversions followed a bias towards a higher frequency of A and very low frequencies of G, C, and T (7∶1∶3∶1). A significant departure from this ratio could indicate documentation or genotyping errors in datasets [54].

The patterns observed for mutational changes in the total sample of patients (Figure 2) were also reproducible when the data were analyzed separately for carriers and non-carriers of nDNA mutations (Supplementary Figure S3).

### Mutational changes in protein mtDNA genes of patients

About 33% of the variants were nonsynonymous, and they were almost homogeneously distributed between the different protein genes (Supplementary Figure S4). There is a quite common misconception in medical genetic studies that tends to interpret nonsynonymous variants as causal mutations by default. It is possible to compare the proportion of nonsynonymous variants in protein genes found in the mtDNA of patients with the proportion observed in healthy individuals. For instance, if we explore the dataset of Coble et al. [55] which consists of 241 complete genomes of mainly European ancestry (mimicking the hg background of our patients), a total of ∼33% of the variation occurred at nonsynonymous positions, as also occurred in the patients.

In our dataset, the ratio of nonsynonymous-synonymous positions in the coding region was about 1∶2.02 (Figure 2); this ratio fits very well with the proportion of 1∶1.97 that was obtained previously [53] in a survey of >5100 entire genomes (see caveats in [56]). According to Kivisild et al. [52]a “*surplus of nonsynonymous mutations is a general feature of the young branches of the phylogenetic tree*” (p.373) [52]. In the entire mtDNAs of patients, although there was an excess of nonsynonymous variants in young branches (*n* = 21; considering young branches to be the terminal ones, only) with respect to the older ones (*n* = 53)(see in Figure 2 “haplogroup defining mutations”), the difference was not statistically significant (Pearson's chi-square test, *p*-value = 0.869); these results are similar to the ones obtained by Pereira et al. [25]. The difference was not statistically significant when considering synonymous-nonsynonmous changes in the nDNA carriers (Pearson's chi-square test, *p*-value = 0.201) and nDNA non-carriers (Pearson's chi-square test, *p*-value = 0.847).

**Figure 3:**
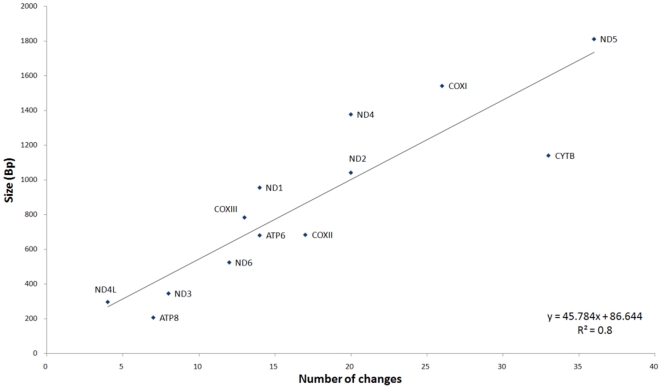
Accumulation of mtDNA changes in the protein genes of patients versus size of the different genes.

**Figure 4:**
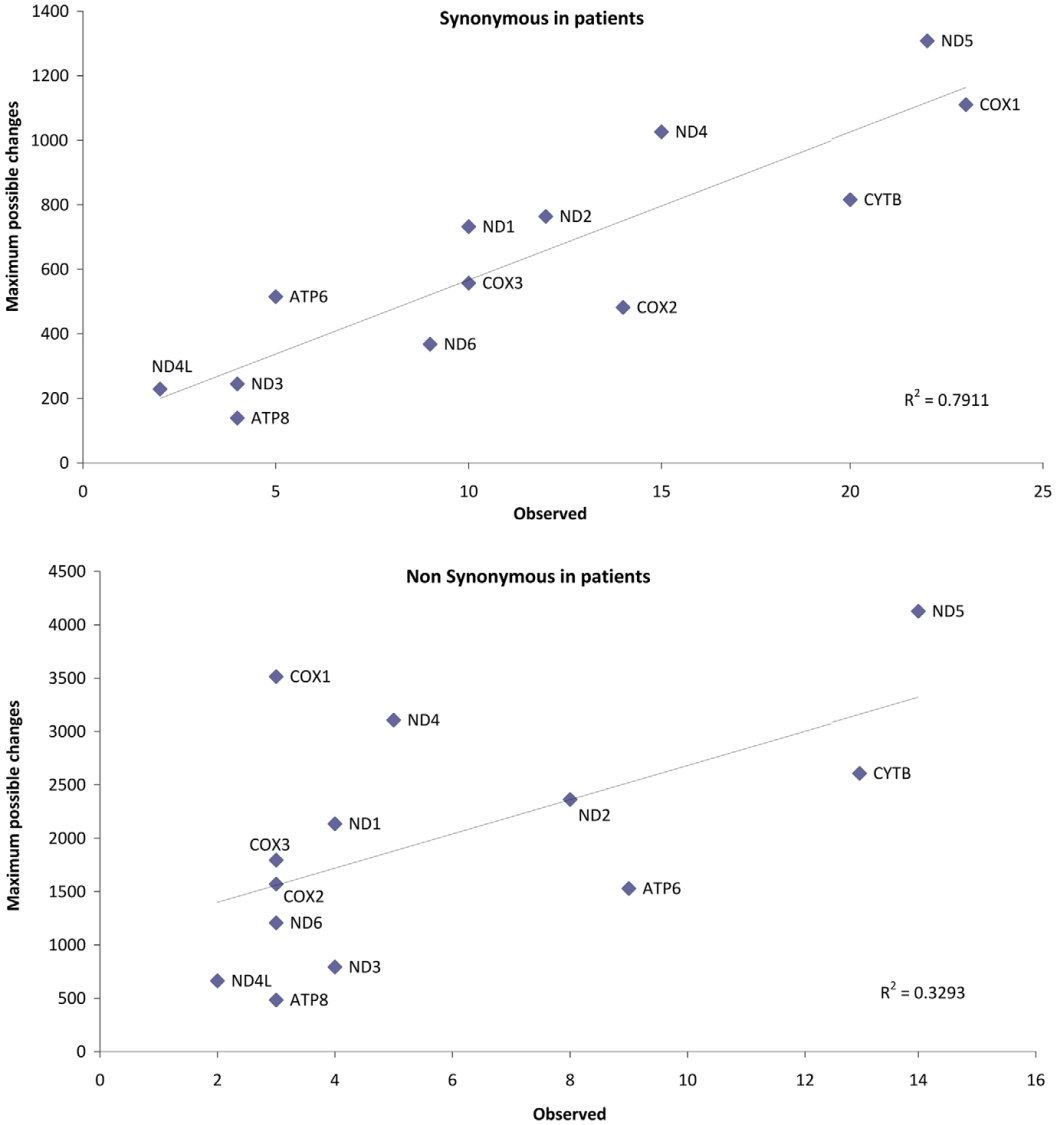
Accumulation of synonymous and nonsynonymous mtDNA changes in the protein genes of patients versus the maximum number of possible changes per gene.

There was a high correlation (R^2^ = 0.8) between the number of changes that accumulated in the mtDNA coding region genes and the length of the gene (Figure 3). This correlation was also evident when only the synonymous changes were considered (R^2^ = 0.79), but not when looking only at the nonsynonymous substitutions (R^2^ = 0.33) (Figure 4). However, the pattern observed for the nonsynonymous changes fits again with the one described for the worldwide phylogeny [53].

The accumulation of replacements per gene followed the same trend regarding the possible maximum number of changes per gene in carriers and non-carriers of nDNA mutations when considering all of the changes together (regarding the length of the genes) and when considering synonymous and nonsynonymous changes separately (Supplementary Figure S5).

### Mutational changes in tRNA mtDNA genes of patients

Mutations in tRNA genes are commonly involved in mtDNA disorders, presumably due to their important role in protein translation. We found ten mutations located in tRNA, most of which were diagnostic of different hgs (Table 3). Some of these mutations are recorded in MITOMAP (http://www.mitomap.org/MITOMAP) as being related to some diseases, although none of them are labelled as “confirmed” pathogenic mutations. Note also that pathogenic indications in MITOMAP have to be considered with caution given the large amount of false positives in the literature and recorded in MITOMAP (see [20], [21]). The most conserved variants in our dataset were the m.12308A>G and m.7561G>A transitions, according to Helm et al. [57]; however, the former is a perfect diagnostic site for the frequent Eurasian haplogroup U and it is unlikely to be responsible for any rare disorder, whereas the latter transition has been previously reported in healthy individuals [58], [59]. The gene that showed the most variants in the mtDNA genomes analyzed was the tRNA^Thr^; this finding also fits well with the prediction of Kivisild et al. [52], indicating that this gene bears significantly more substitutions than any other when observing the worldwide phylogeny. Although some of the tRNA mutations seem to be evolutionarily well conserved, none of them have a pattern of segregation with the disease, and none have been confirmed as pathogenic in the literature. Taking all these results together, tRNA mutations do not seem to play a pathogenic role in NS or related disorders [60].

**Table 3:** Variants observed at the mtDNA tRNA genes of Ras/MAPK pathway syndromes patients.

Mutation position	Sample ID^1^	Nucleotide change	tRNA	Location in secondary structure	Hg	MITOMAP	Conservation^2^
4336	#13, #14	T-C	tRNA-Gln	Acceptor stem	H5a, U6d	ADPD/hearing loss & migraine (unclear)	50%<x<90%
7476	#4*	C-T	tRNA-Ser^UCN^	Anticodon stem	J2	Not reported	50%<x<90%
7521	#44, #45*	G-A	tRNA-Asp	Acceptor stem	L3′4′6, G4, M76	Not reported	Different in human and mammalian consensus
7561	#39*	T-C	tRNA-Asp	Variable loop	-	Not reported	90%<x<100%
10463	#5*, #6	T-C	tRNA-Arg	Acceptor stem	T, J1c1b1a, P4a	Not reported	50%<x<90%
12285^3^	#45*	T-G	tRNA-Leu^CUN^	DHU loop	L2a5	Not reported	50%<x<90%
12308	#33*, #34; #35, #36, #37*, #38*, #39*, #40	A-G	tRNA-Leu^CUN^	Variable loop	U	CPEO/stroke/CM/renal & prostate cancer risk/altered brain pH	100%
15904	#17, #18, #19	C-T	tRNA-Thr	DHU loop	HV0a	Not reported	Natural variable site
15927	#43	G-A	tRNA-Thr	Anticodon stem	X2b, B5b, U6a5, L0f2b, G3b, HV1a1	Multiple sclerosis/DEAF1555 increased penetrance (P.M/possible helper mutation)	Different in human and mammalian consensus
15928	#5*, #6	G-A	tRNA-Thr	Anticodon stem	T, L3x2b, C7b, Z3a, M25, M35b	Multiple sclerosis (P.M)	50%<x<90%

NOTE.

1 Starst identified samples carrying nDNA mutations;

2 According to Helm et al. [57];

3 Transversion 12285T>G is not actually a private variant if we consider that a new branch, L2a5, has been defined in the present article based on this entire genome and another one previously described in the literature under the GenBank entry HM596745.

### Recurrent mutations in mtDNAs of patients

As indicated in Table 2, the homoplasmic mutations did not concentrate in particular hgs or genes; some of them were found at the tips of the phylogeny (see next section: private variants). Most of these mutations are well-known hotspots in the phylogeny [44], with the only exception of m.4674A>G that appears as a private substitution in two patients and which received two hits in Soares et al. [44] (Table 2). The apparent overrepresentation of tRNA and nonsynonymous mutations among recurrent mutations was previously observed [52] for the worldwide mtDNA phylogeny (Table 2).

### Private mutations in mtDNAs of patients

Some other variants observed in our cases were private (Table 4) if we consider the private changes regarding their status in the phylogeny of Figure 1 (mutations located at the terminal branches). Nonsynonymous changes were more common among private variants (nonsynonymous:synonymous ratio: 1∶1.91) than within haplogroup defining mutations (1∶2.08); this is because natural selection had more time to filter out deleterious changes from the older branches than the younger branches (see above).

**Table 4:** Private coding region mutations observed in the entire mtDNA genomes of the patients (see Figure 1) that are “novel” or are recorded in MITOMAP as (confirmed or unconfirmed) disease-associated variants.

Positions	Sample ID^1^	Location	Nucleotide change	Synonymous/nonsynonymous(aa change)	Haplogroup^2^	mtDNA Mutations with reports of disease-associations in MITOMAP^3^
827	#33*	*12s rRNA*	A-G	–	G1a1a1, D4h1a2, R0a1, B4b’d’e	Maternally inherited deafness or aminoglycoside-induced deafness (conflicting reports-B4b'd marker)
961	#33*	*12s rRNA*	T-C	–	M7a2b, M44, D4h2, N9a2, A5b, R6a1a, B2i, U5a1c2, U4a1a, L0a1b1a1, L6, M2a1a2a1a	Maternally inherited deafness or aminoglycoside-induced deafness/possibly left ventricular non-compaction-associated (unclear)
1820	#1	*16s rRNA*	A-G	–	‘Novel’	Novel
4796^*4^	#36	*ND2*	C-T	Synonymous	–	Novel
5029	#5*	*ND2*	T-C	(M/T) Neutral apolar-neutral polar	‘Novel’	Novel
5911	#11*	*COX I*	C-T	(A-V) Neutral apolar-neutral apolar	R8a1, L0a1b	Prostate cancer (reported)
8544	#44	*ATP6*	C-T	Synonymous	‘Novel’	Novel
8544	#44	*ATP8*	C-T	(S-L) Neutral polar-neutral apolar	‘Novel’	Novel
10081^4^	#18	*ND3*	T-C	(M/T) Neutral apolar-neutral polar	–	Novel
10205	#7	*ND3*	C-T	Synonymous	‘Novel’	Novel
11026	#17	*ND4*	A-G	Synonymous	‘Novel’	Novel
11778^5^	#40	*ND4*	G-A	(R-H) Basic polar-basic polar	–	LHON (confirmed); progressive dystonia (confirmed)
12103A^4^	#36	*ND4*	C-A	Synonymous	–	Novel
14502	#33*	*ND6*	T-C	(I-V) Neutral apolar-neutral apolar	M10, X2a, R8b2, P7, N11a	LHON (reported-possible synergistic)
14668	#19	*ND6*	C-T	Synonymous	Z2, D4, L5a1b	Major depressive disorder-associated (reported)
14831	#29	*CYTB*	G-A	(A-T) Neutral apolar-neutral polar	L1c3b2	LHON (reported)
15175	#14	*CYTB*	C-T	Synonymous	M9a1a1d	Novel

NOTE.

1 Starst identified samples carrying nDNA mutations;

2 Mutations defining haplogroup(s) according to Phylotree and the data obtained here; “novel” means a variant that was not found in Phylotree [36], mtDB [62], HmtDB [63], and Google searches as executed in [20], [22];

3 The ‘novel’ condition is as indicated in MITOMAP;

4 Note that m.4796C>T and m.12103C>A were reported by Gasparre et al. [64] as novel changes in oncocytoma and CCRCC, m.4796C>T pop-up in HmtDB as reported by Porcelli et al. [65], although this variant does not appear in the original publication, and m.10081T>C appears in Zheng et al. [66] but as generated by human pol γ in vitro;

5 m.11778G>A is a well-confirmed mutation responsible for LHON and progressive dystonia, and, it fact, this pathogenic mutation appeared in a NS patient who also suffered from LHON (see Figure 1, #40); aa: amino acid.

Most of the private variants had already been reported in the literature in healthy individuals, with some of them appearing sporadically in different hg backgrounds. Some private variants were even reported as possibly pathogenic in MITOMAP, but this was never confirmed, with the exception of m.11778G>A, a well-known mutation responsible for Leber hereditary optic neuropathy LHON and progressive dystonia (patient #40; Table 4). In addition, all of these variants appear simultaneously as polymorphisms in MITOMAP (obviously with the exception of m.11778G>A). Only some of the variants listed in Table 4 are actually private and were referred to here as “novel”, in the understanding that “novel” means a variant that could not been found in the main mtDNA databases and does not show up on Google searches (Table 4). This “novel” condition alone cannot be used to attribute a causal role to these variants; in fact, any dataset of either healthy or unhealthy individuals will contain an large proportion of private variants, even taking into account the fact that there are more than 8700 complete or semi-complete genomes available in the literature to date (http://www.phylotree.org/). For instance, in this large dataset of entire complete genomes, more than 60% of the transversions and 28% of the transitions were only observed once (private substitutions). Taking all of this evidence together, it seems unlikely that any of the private variants observed in the patients are involved in the NS or Noonan-like phenotypes.

### Heteroplasmic variants in mtDNAs of patients

Quite often, common mtDNA diseases are related to mutations with a heteroplasmic status. Six different heteroplasmies were found in the 45 patients analyzed (Table 5). Two of them fell in the control region and were highly recurrent in the phylogeny, especially position 16093 [44]. Only one of the positions, 15924, fell in the tRNA^Thr^, but this is also a well-known mtDNA hotspot. Patient #11 carried two heteroplasmic variants (positions 4992 and 5144), but both were synonymous changes on gene *ND2*. Another position (10784) fell in the *ND4* gene and was a nonsynonymous variant that changed the amino acid isoleucine to valine, but it appeared in a healthy individual belonging to haplogroup U6a1b (GenBank accession number: EF064320).

**Table 5:** Features of heteroplasmic variants found in patients.

Position	Heteroplasmy	rCRS	Loci	Sample ID	GenBank and/or otherdatabase searches (hg)	Soares et al.Score
4992	G>A	A	ND2	#11	AP010974 (D4b2b1)	0
5144	C>T	C	ND2	#11	–	0
10784	G-A	A	ND4	#9	EF064320 (U6a1b)	1
15924	A>G	A	tRNA^Thr^	#15, #16	Common polymorphism	30
16286	T>C	C	D-loop	#37	Common polymorphism	5
16093	T-C	T	D-loop	#32	Common polymorphism	79

## Discussion

The patients analyzed in the present study (together with other related disorders) represented the largest cohort of patients analyzed to date by far for variations in the mtDNA molecule. The analysis of entire, complete genomes has, for the first time, enabled the implementation of an evolutionary approach aimed at discovering the potential pathogenicity of mtDNA changes in patients. The known human mtDNA phylogeny is based on 8731 complete mtDNA genomes (Phylotree), and, therefore, it provides a solid background to compare the variation observed in the mtDNA genomes of patients against.

Analysis of the data showed that the pattern of mutations in tRNA genes and the pattern of nonsynonymous changes in protein genes fit well with the variation observed in natural human populations. In other words, replacements, substitutions and tRNA mutations are not more prevalent in the mtDNA of patients than expected. In addition, most of the nonsynonymous mutations that were observed in the genomes of patients are common polymorphisms widely distributed throughout the global mtDNA tree. The recurrent and private mutations inferred from the phylogeny of entire mtDNAs from patients are also as expected, according to the patterns observed in human populations. Heteroplasmic mutations were also rare among the patients, and the few found did not seem to play a pathogenic role given their presence in healthy individuals too. Therefore, the evolutionary view of entire mtDNA genomes of patients does not support a role of mtDNA variants in the NS phenotype or in Ras/MAPK pathway syndromes. Moreover, the pattern observed for carriers of nuclear DNA mutations was very similar to that of non-carriers (Supplementary Figures S1, S2, S4, and S5).

In addition, there was no prevalent mutation in our patients nor a hg background apparently associated with the clinical phenotypes. Therefore, the theory of a highly penetrant mtDNA mutation being responsible for NS or Ras/MAPK pathway syndromes can be completely disregarded by the data obtained in the present study. In contrast to the Mendelian-like dominant pattern observed in most Ras/MAPK pathway syndromes cases (involving nDNA mutations), one could alternatively hypothesize a multi-factorial (genetic) complex scenario where some phenotypes could be explained by the sum (or interactions) of small effects contributed by different nuclear and/or mtDNA genes. Although the evolutionary approach employed here does not reveal the existence of a predominant variant in patients, a population-based approach (e.g. case-control study) could be used instead to reveal the existence of such low risk mtDNA variants. However, such an approach would need proper population sample sizes (in order to obtain reasonable power to detect any positive associations, e.g. 80%), monitorization of population stratification (which is particularly problematic in mtDNA studies) [27], [61], and adequate corrections for multiple tests, amongst others. Deficient study designs or wrong statistical treatments of the data could easily lead to false positives of an association. With this in mind, one could retrospectively look to the previous evidence suggesting the weak association between mtDNA variants and NS. The case-control study by Castro et al. [23] represents a paradigmatic example. These authors genotyped eight mtDNA variants in 130 Spanish HCM patients and 300 healthy controls; note that HCM is one of the characteristic phenotypes in NS patients (Table 1). According to the authors, “*Because multiple comparisons were taken into account (9 haplogroups and 8 SNPs), we used the Bonferroni*'*s correction and a p<0.01 was considered as the level of statistical significance.*” It is not clear from the text whether the *p*-value mentioned refers to the initial nominal value or the one adjusted using Bonferroni. Either way, if one assume an standard nominal significant value of *α* = 0.05, an adjusted *p*-nominal value using Bonferroni for 17 independent tests (mtSNPs) would lead to a threshold for significance of 0.0029 (but not 0.01). However, Castro et al. claimed to have found a positive association between hg T (variant G13368A) and HCM, supported by a *p*-value = 0.007, which is above the correct adjusted nominal value.

Recently, Rani et al. [24] analyzed the complete genome of seven NS patients lacking *PTPN11* mutations. They found that all of them belonged to different sub-lineages of hg R (including R7b1b, R30a1, R30c, T2b7, and U9a1), but the common factor in all of them was the lack of transitions at positions 12705 and 16223 (that lead from hg N to hg R). Since the authors only screened their patients for mutations at *PTPN11* (which, worldwide, explains about 50% of the NS cases), their patients could have carried mutations at any of the other genes commonly held responsible for NS [2]. The study by Rani et al. [24] does not explain why the transitions at 12705 and 16223 should be responsible for the NS condition. Note that hg R represents the most common macro-hg in Europe (e.g. ∼92% in northern Iberia [28] and ∼87% in our cohort of northern Iberian patients; Pearson's chi-square test, *p*-value = 0.939); and that it includes the macro-hg R0 (where hg H is nested), hg U, hg J and T, amongst others. Therefore, the observations by Rani et al. contradict the scenario observed for our European patients: (i) our cases showed a lower frequency of hg R than a typical control group (although the difference was not statistically significant), and (ii) the supposed pathogenic variants (the ones defining hg R) are the predominant ones in Europe, an observation that is difficult to reconcile with the prevalence of NS worldwide. As also mentioned by Rani et al., India is very complex genetically; this means that any study aiming to evaluate the association between mtDNA variants and any disease should take the confounding effect of population sub-structure into account. Finally, Rani et al. deliberately considered hg R to be R minus U : “*…followed by hgs R and U with frequencies of 14.27%, 15.23%, respectively…”*) (p. 169) [24] and minus T (see Table 4 and Figure 3 in Rani et al. [24]) in the controls, but they included U and T within R in cases (as one of their patients belonged to hg U9a1 and another one to T2b7), and thereby they artificially created more differences in the apparent frequency between the samples than was actually the case. Moreover, apart from U and T, their R category should also have included the controls who belonged to hgs H2 and J1b, because all of them shared the feature common to the rest of the sub-lineages of hg R, which is the lack of mutations at positions 12705 and 16223. Finally, independent of possible population stratification, any case-control study based on seven cases and 105 controls has a very low *a priori* power for detecting a positive statistical association when the risk effect being looked for is weak. Therefore, their main conclusion “*The haplogroup R by itself may be susceptible to disease phenotype or different environmental background or some of the unidentified nuclear gene might render susceptibility to disease phenotype…*” (p. 171) [24] have little support in view of the contradictions mentioned above.

On the other hand, the results and conclusions of the studies by Dhandapany et al. [18] and Prasad et al. [19] were also critically questioned by Bandelt et al. [21] based on two main arguments: (i) their “novel” mutations were, in reality, not novel at the time of publication, and (ii) there is little support in favour of the causal role of these mutations in NS because most of them (if not all) are common polymorphisms, for example, the transition of m.2755A>G characterize hg R8. Our results agree with the conclusions of Bandelt et al. [21]: that authors tend to overstate the novelty of particular mtDNA variants and impute them a pathogenic role based on this “novel” condition. In reality, most of these variants were polymorphisms already known and which are unlikely to constitute pathogenic mutations. In case-control association studies, spurious positive associations generally show up when using deficient statistical approaches, or when under the presence of the population stratification, which is particularly problematic in mtDNA association studies because its reduced effective population sizes in comparison to average nuclear genes.

### Conclusions

The analyses of replacement substitutions and other variants observed in the patients suffering Ras/MAPK pathway syndromes (tRNA, private, recurrent and heteroplasmic mutations), as well as the pattern of hg frequencies indicated that this variation can be fully expected as in any typical European dataset. Changes in mtDNA genomes of patients are therefore unlikely to be related to NS phenotype or Ras/MAPK pathway syndromes. The combined evolutionary and phylogeographic approach employed here seems more appropriate for evaluating the potential pathogenicity of mtDNA variants than a case-control study when the risk effect and the sample size are too low to provide reasonable statistical power or when it is under the presence of a population sub-structure.

## Supporting Information

Figure S1 Haplogroup frequencies in the patients and in a typical Iberian sample of healthy individuals [28]. For the sake of clarity, some macro-haplogroups were sub-divided into main sub-haplogroups and other aggregated paragroup categories (e.g. phylogenetically, hg R0 should be considered as the sum of H+V+other-R0; and U should be considered as the sum of U5+K+other-U); the phylogenic relationships are clarified in Figure 1 and, more generally, in the worldwide phylogeny of Phylotree.
(TIF)

Figure S2 Distribution of synonymous and nonsynonymous changes in the mtDNA protein genes of all patients, and also considering carriers and non-carriers of nuclear mutations separately.
(TIF)

Figure S3 Number of different types of amino acid changes regarding nonsynonymous substitutions.
(TIF)

Figure S4 Summary of the main features regarding different types of mtDNA changes in the patients divided into carriers and non-carriers of nDNA mutations.
(TIF)

Figure S5 For carriers and non-carriers of nDNA mutations: accumulation of mtDNA changes in protein genes versus the size of the different genes, and accumulation of synonymous and nonsynonymous mtDNA changes in the protein genes versus the maximum number of possible changes per gene.
(TIF)

Table S1 Mitochondrial DNA variants observed in the 45 entire complete genomes of the patients. The notation of the variants is explained in the legend of Figure 1. Heteroplasmic positions were also included.
(XLS)
