# Supplementary figures and images for: Evolutionary Analyses of Entire Genomes Do Not Support the Association of mtDNA Mutations with Ras/MAPK Pathway Syndromes
Source: PLoS One. 2011 Apr 19;6(4):e18348. doi: 10.1371/journal.pone.0018348 (PMC3079712; doi:10.1371/journal.pone.0018348)

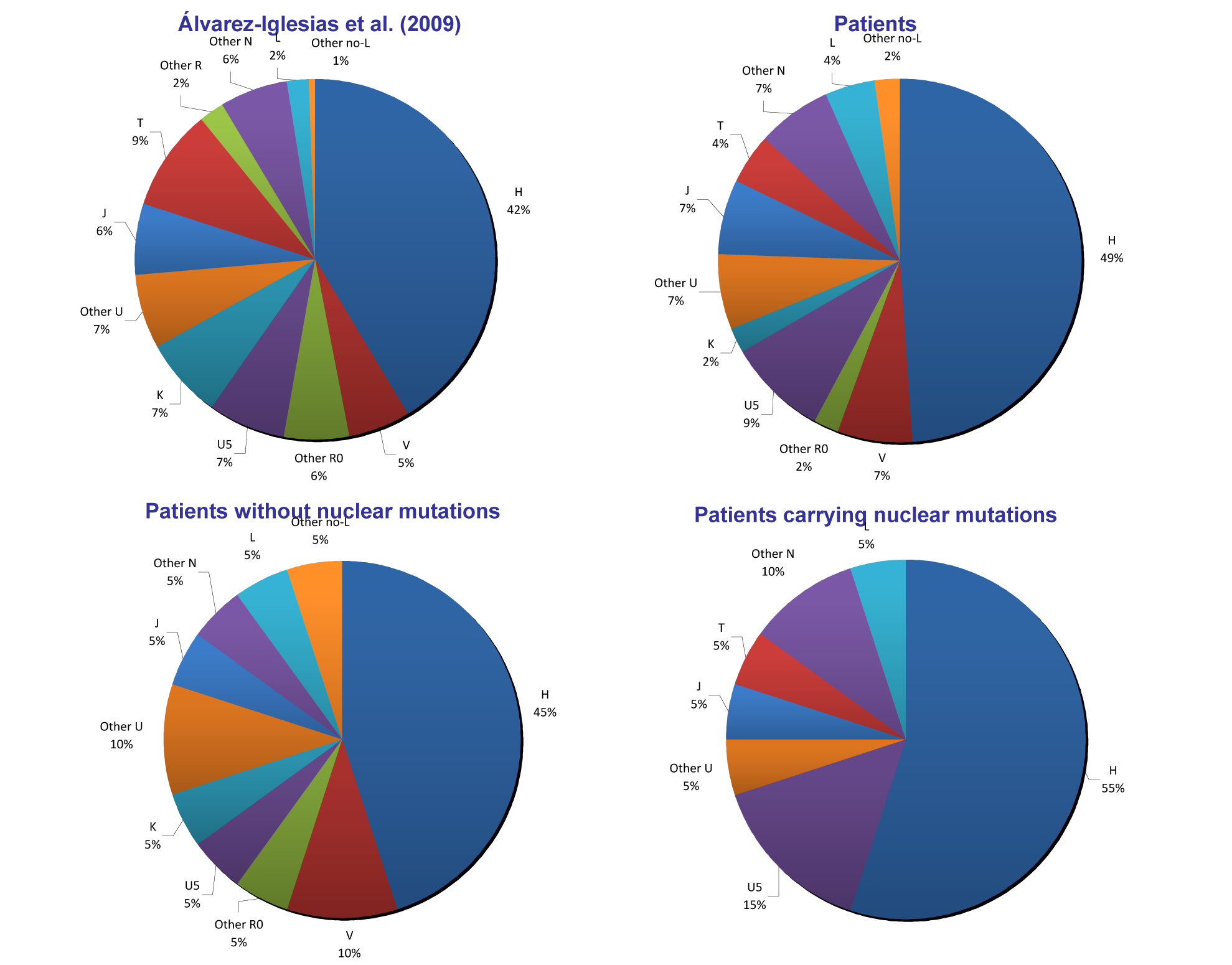

Supplement: Figure S1 — Haplogroup frequencies in the patients and in a typical Iberian sample of healthy individuals [28]. For the sake of clarity, some macro-haplogroups were sub-divided into main sub-haplogroups and other aggregated paragroup categories (e.g. phylogenetically, hg R0 should be considered as the sum of H+V+other-R0; and U should be considered as the sum of U5+K+other-U); the phylogenic relationships are clarified in Figure 1 and, more generally, in the worldwide phylogeny of Phylotree. (TIF) [file pone.0018348.s001.tif]

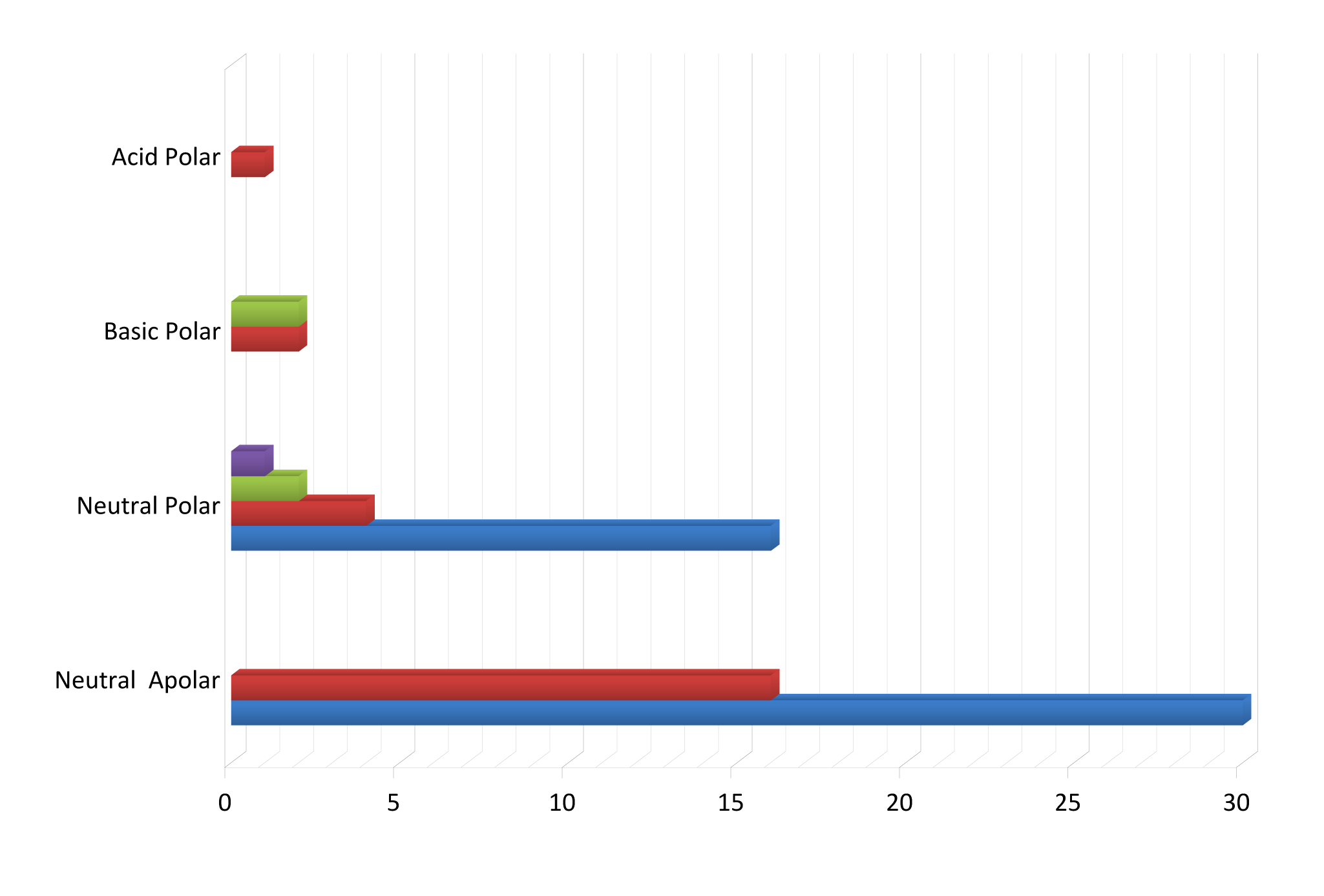

Supplement: Figure S2 — Distribution of synonymous and nonsynonymous changes in the mtDNA protein genes of all patients, and also considering carriers and non-carriers of nuclear mutations separately. (TIF) [file pone.0018348.s002.tif]

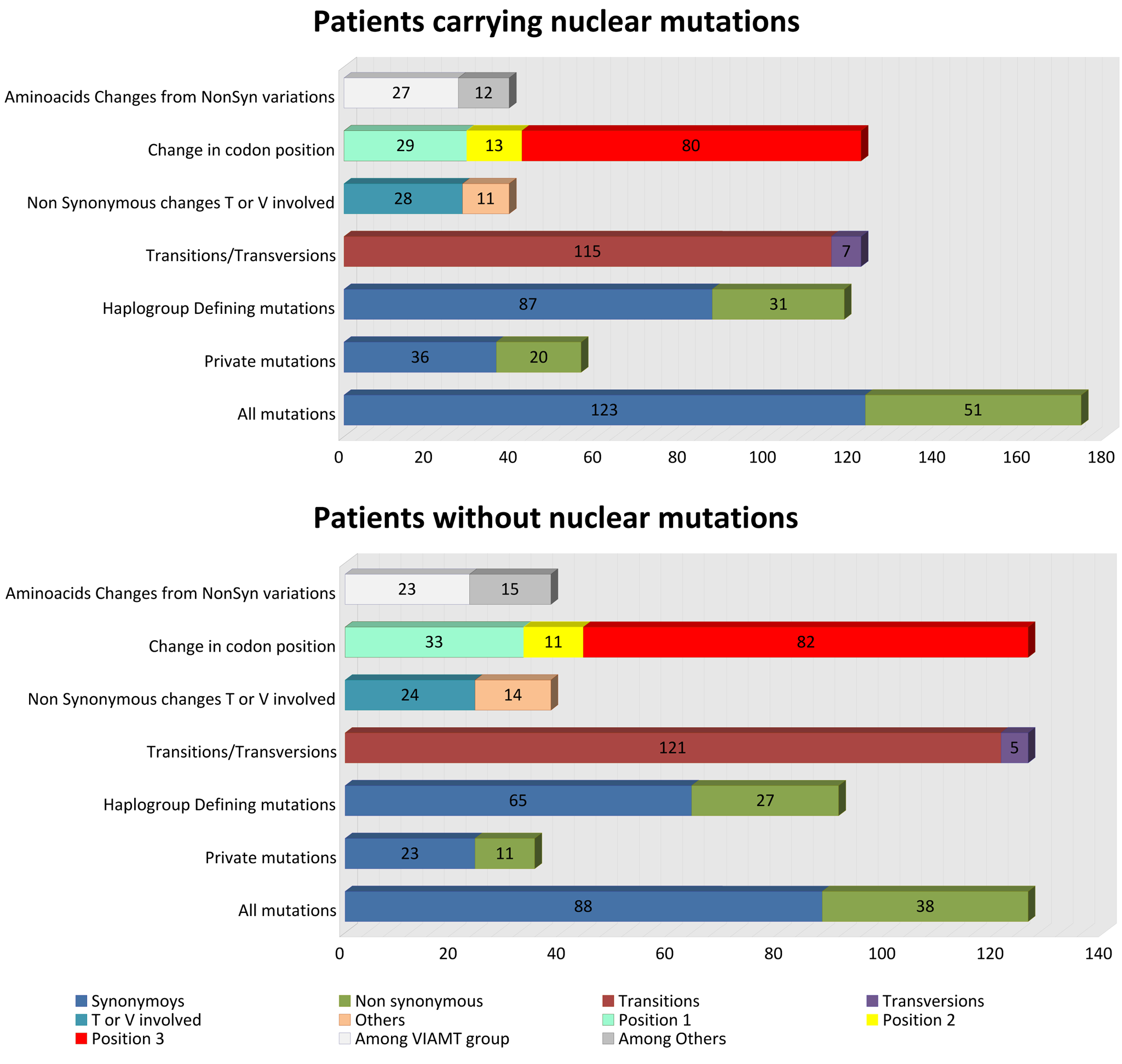

Supplement: Figure S3 — Number of different types of amino acid changes regarding nonsynonymous substitutions. (TIF) [file pone.0018348.s003.tif]

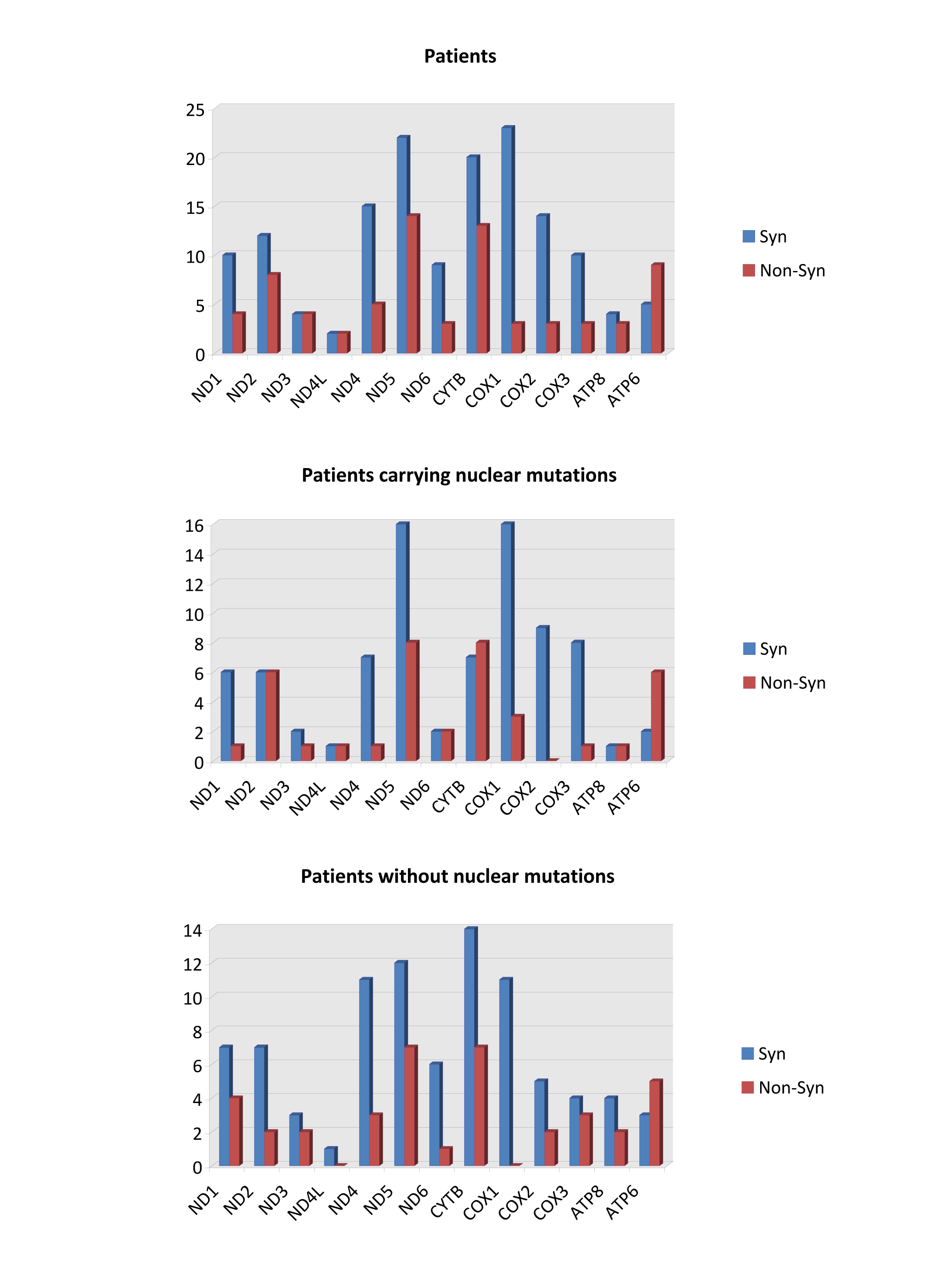

Supplement: Figure S4 — Summary of the main features regarding different types of mtDNA changes in the patients divided into carriers and non-carriers of nDNA mutations. (TIF) [file pone.0018348.s004.tif]

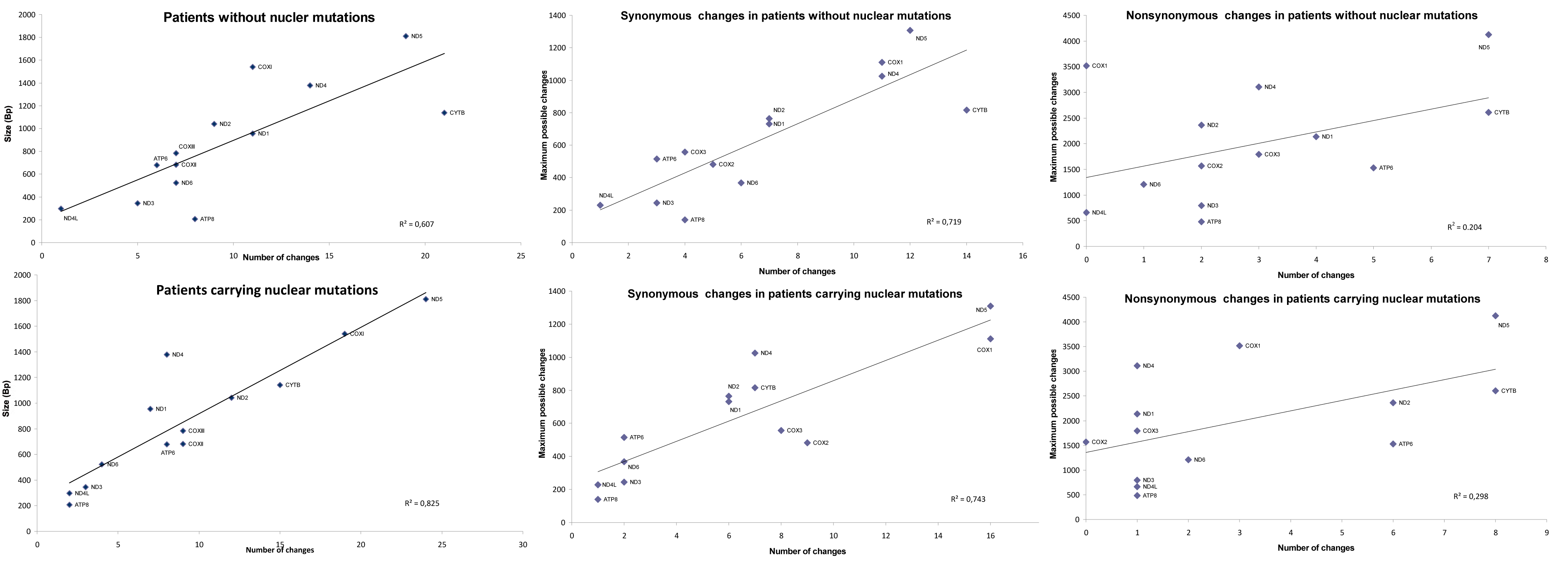

Supplement: Figure S5 — For carriers and non-carriers of nDNA mutations: accumulation of mtDNA changes in protein genes versus the size of the different genes, and accumulation of synonymous and nonsynonymous mtDNA changes in the protein genes versus the maximum number of possible changes per gene. (TIF) [file pone.0018348.s005.tif]
